# Transcriptome Profiling of Starvation in the Peripheral Chemosensory Organs of the Crop Pest *Spodoptera littoralis* Caterpillars

**DOI:** 10.3390/insects12070573

**Published:** 2021-06-23

**Authors:** Erwan Poivet, Aurore Gallot, Nicolas Montagné, Pavel Senin, Christelle Monsempès, Fabrice Legeai, Emmanuelle Jacquin-Joly

**Affiliations:** 1 Institute of Ecology and Environmental Sciences of Paris, INRAE, Sorbonne Université, CNRS, IRD, UPEC, Université de Paris, 78000 Versailles, France; erwanpoivet@gmail.com (E.P.); aurore.gallot@univ-lyon1.fr (A.G.); nicolas.montagne@sorbonne-universite.fr (N.M.); christelle.monsempes@inrae.fr (C.M.); 2 IRISA, INRIA, CNRS, Université de Rennes, 35000 Rennes, France; seninp@gmail.com (P.S.); fabrice.legeai@inrae.fr (F.L.); 3 IGEPP, INRAE, Institut Agro, Université de Rennes, 35000 Rennes, France

**Keywords:** starvation, olfaction, RNA-seq, caterpillars, *Spodoptera littoralis*

## Abstract

**Simple Summary:**

Starvation increases olfactory sensitivity in a manner that enhances the search for food in animals, including insects. However, the molecular mechanisms via which starvation modulates olfactory receptor neuron function are poorly understood. In this study, we sequenced and compared the whole transcriptomes of the main olfactory organs (antennae and palps) of fed and starved caterpillars from the species *Spodoptera littoralis*. We revealed that transcripts involved in several biological processes are regulated upon starvation. These processes include glucose metabolism, immune defense, foraging activity, and olfaction. In this last process, we evidenced regulation of chemosensory proteins and odorant-degrading enzymes, known to play a role in the dynamics and the sensitivity of the olfactory receptor neuron response. Our results identify new elements in the cascade of olfactory neuron modulation, in addition to insulin, GABA, and short neuropeptide F signaling.

**Abstract:**

Starvation is frequently encountered by animals under fluctuating food conditions in nature, and response to it is vital for life span. Many studies have investigated the behavioral and physiological responses to starvation. In particular, starvation is known to induce changes in olfactory behaviors and olfactory sensitivity to food odorants, but the underlying mechanisms are not well understood. Here, we investigated the transcriptional changes induced by starvation in the chemosensory tissues of the caterpillar *Spodoptera littoralis*, using Illumina RNA sequencing. Gene expression profiling revealed 81 regulated transcripts associated with several biological processes, such as glucose metabolism, immune defense, response to stress, foraging activity, and olfaction. Focusing on the olfactory process, we observed changes in transcripts encoding proteins putatively involved in the peri-receptor events, namely, chemosensory proteins and odorant-degrading enzymes. Such modulation of their expression may drive fluctuations in the dynamics and the sensitivity of the olfactory receptor neuron response. In combination with the enhanced presynaptic activity mediated via the short neuropeptide F expressed during fasting periods, this could explain an enhanced olfactory detection process. Our observations suggest that a coordinated transcriptional response of peripheral chemosensory organs participates in the regulation of olfactory signal reception and olfactory-driven behaviors upon starvation.

## 1. Introduction

The sense of smell and food intake are strongly connected in animals. Olfaction is indeed a critical regulator of food-seeking behaviors across species, and food odor alone can trigger behaviors essential for survival [1,2]. For example, in humans, smelling dark chocolate without eating it increases the feeling of satiety [3]. In rats, food deprivation increases olfactory abilities [4]. In mammals, food intake is under the control of two major anorectic hormones, leptin and insulin [5]. These hormones have been shown to regulate olfactory behaviors, targeting both the central and the peripheral olfactory systems [6,7,8]. In insects, the nutritional status—satiety or hunger—also regulates olfactory sensitivity. After a blood meal, mosquitoes show a decreased attraction toward prey odors [9,10,11], changes in olfactory sensitivity [11], and down-regulation of some chemosensory genes [10,12,13]. Olfactory behavior learning is not possible with fed insects [14,15]. Conversely, upon starvation, fruit flies exhibit an increase in their behavioral response to different odors including food odors [16,17], a modification in pheromone perception and courtship [18], and a reduced behavioral avoidance to aversive odors [19].

Several studies investigated the mechanisms via which starvation affects odor-guided behaviors in insects. Electrophysiological recordings conducted on *Drosophila melanogaster* antennae revealed no difference in responses of an odorant receptor (OR47a) to 3-methylthio-1-propanol in the fed or fasted state, whereas an increased olfactory attraction to this compound was observed upon starvation [16]. The authors, thus, suggested that the increased behavioral attraction in the starved state may be caused by more central mechanisms, such as increased activity in the antennal lobe—the primary olfactory processing center—or higher brain structures [16]. However, other studies conducted at the periphery on a variety of *Drosophila* olfactory receptor neuron (ORN) types did not support this hypothesis since an increased physiological activity was observed upon starvation in all cases [17]. In triatomines, electroantennography also revealed modulation of the peripheral sensory responses upon starvation [20]. In *Drosophila*, the short neuropeptide F (sNPF) that is known to be a modulator of nutrient sensing is expressed in ORNs [21]. Neural signaling mediated by sNPF was shown to increase olfactory-mediated food-seeking behavior after starvation in flies [22]. In this later study, starvation was shown to increase sNPF receptor signaling, which in turn enhanced presynaptic activity in *Drosophila* olfactory neurons. It has also been shown that insulin signaling interacts with GABA signaling to impact ORN function upon starvation in *Drosophila* [23], potentially impacting the expression of downstream genes. Thus, modulation of the peripheral olfactory activity makes an important contribution to food search behavior in response to starvation.

At the molecular level, several studies have investigated the transcriptional changes induced by starvation using microarray approaches on *Drosophila* [24,25] or RNA sequencing (RNA-seq) [26], but only a few precisely focused on the chemosensory organs [16,27]. Zinke et al. [24] investigated the whole body of larvae, while Fujikawa et al. [25] focused on the adult heads, with both studies searching for genetic mechanisms underlying nutrient signaling. Farhadian et al. included chemosensory tissues, namely, antennae, maxillary palps, and proboscis [16], and Ko et al. used adult antennae [27]. Apart from this last study that focused only on G-protein-coupled receptor transcript variation, reasonable correspondence could be evidenced across the different studies, such as downregulation of genes involved in immunity, response to stresses, and/or detoxification and resource allocation upon starvation, as well as the regulation of several chemosensory genes, including odorant-binding proteins (OBPs). Interestingly, the nutrient level has also been shown to regulate circadian oscillating genes [25], including the potential clock gene *takeout* [16]. Takeout proteins are known to be involved in foraging activity regulation and in improving the sensitivity of taste nerves to carbohydrates during starvation [28].

These different studies were all conducted in *Drosophila*, a convenient genetic model organism for which a whole genome array is available. However, we are still far from understanding the molecular events that occur in the peripheral olfactory organs upon starvation. The use of other insect models will help in identifying common gene families modulated in response to starvation and, thus, allow proposing common processes underlying enhanced olfactory responses. Today, RNA-seq approaches have proven to be very efficient for transcriptome expression studies [29,30,31], allowing the rapid development of a variety of new insect models. *Spodoptera littoralis*, a noctuid moth, is one of these new model species [32]. The study of the feeding behavior of this herbivorous species is particularly relevant since, as many Lepidoptera, it is an important crop pest. A better understanding of the mechanisms that regulate olfactory sensitivity and, thus, food intake, offers the possibility to develop olfactory-based strategies to perturb these critical behaviors. For instance, as early as in 1985, Cain et al. [33] showed in field experiments that 20 h starved caterpillars of the crop pest *Pieris rapae* were more efficient in localizing their host plant than fed caterpillars. Here, we investigated the transcriptional changes induced by starvation in the peripheral chemosensory tissues of *S. littoralis* caterpillars, using RNA-seq expression profiling. We took advantage of a reference transcriptome we previously established in this species [32,34,35], in which we annotated 127 chemosensory genes, to align and count Illumina reads obtained from antennae and maxillary palps collected from either fed or 24 h starved caterpillars. This led us to identify 81 transcripts whose expression levels are modulated according to nutritional status, including chemosensory genes. Our results contribute to a better understanding of the peripheral mechanisms linked to food intake.

## 2. Materials and Methods

### 2.1. Insect Rearing and Behavioral Assay

**Figure 1:**
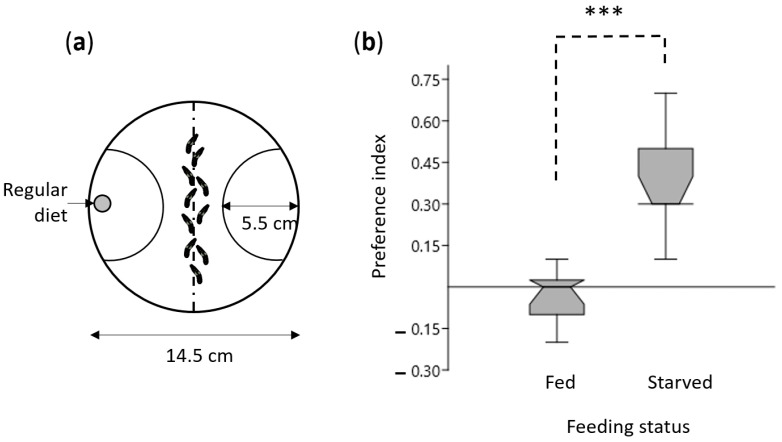
Starvation enhances olfactory behavior: (**a**) The choice test was performed in a closed glass Petri dish (14.5 cm diameter). One regular diet spot (1 g) was deposited on one side of the dish. Ten L4 larvae were placed in the middle of the dish. The dish area was divided into two parts delimited by arc circles centered on the food spots (radius 5.5 cm). The numbers of larvae in each area were computed at 10 min; (**b**) Box plots showing the median and the first and third quartiles of the distribution. Bars delimitate maximum and minimum values. The preference indices were calculated as follows: (number of larvae in the diet area—number of larvae in the empty area)/total number of larvae; *n* = 10; *** *p* < 0.001, Mann–Whitney test.

*S. littoralis* were reared in the laboratory on a semiartificial diet [36] at 22 °C, 60% relative humidity, under a 16 h/8 h light/dark cycle. At the fourth larval instar (L4), some larvae were deprived of food in the middle of their photophase, while others were kept on the diet. The behavioral tests were conducted 24 h later (middle of the next photophase) in closed glass Petri dishes (14.5 cm diameter) under red dim light. One regular diet spot (1 g) was deposited on one side of the dish (Figure 1a). Ten L4 larvae were placed in the middle of the dish. The dish area was divided into two parts delimited by arc circles centered on the food spots (radius 5.5 cm). The numbers of larvae in each area were computed after 10 min, and preference indices were calculated as follows: (number of larvae in the diet area—number of larvae in the empty area)/(total number of larvae). Ten independent replicates, each including 10 larvae, were performed, and preference indices were compared using a Mann–Whitney test (Figure 1b). Each caterpillar was tested only once.

### 2.2. Tissue Collection and Illumina Sequencing

At the fourth larval instar, some larvae were deprived of food in the middle of their photophase, while others were kept on the diet. Then, 24 h later, antennae and maxillary palps were dissected from ~100 starved larvae (SLAP: starved larvae antennae and palps) and ~100 fed larvae (FLAP: fed larvae antennae and palps). We performed all dissections at the same time-point (middle of the photophase) for both fed and starved larvae to avoid any circadian rhythm effect on gene expression. This operation was reproduced three times on three different generations to avoid any generation effect. Total RNAs were extracted from each sample using TRIzol^®^ Reagent (Invitrogen, Carlsbad, CA, USA). Then, RNAs from starved larvae were pooled together, as were RNAs from fed larvae. These two RNA samples were independently used as templates for cDNA synthesis and Illumina sequencing as described in [32] (HiSeq2000, one channel for the two samples, single read; GATC Biotech SARL). Totals of 1,947,899 and 2,389,809 raw reads were obtained from FLAP and SLAP samples, respectively (Table 1). The RNA-seq data were deposited in GenBank (BioProject) under the accession numbers SAMN01908929 (fed *S. littoralis* larvae) and SAMN01908927 (starved *S. littoralis* larvae).

### 2.3. Illumina Read Alignment and Statistical Analysis

Processed Illumina datasets FLAP and SLAP were aligned on the *S. littoralis* reference transcriptome [32] with Bowtie [37]. Totals of 1,513,384 out of 1,807,931 processed sequences (FLAP) and 1,813,092 out of 2,171,664 sequences (SLAP) were successfully aligned, representing ratios of 83.7% and 83.5% of the trimmed datasets, respectively (Table 1). Unaligned reads and multiple aligned reads were excluded from further analyses. For both FLAP and SLAP datasets, the number of reads aligned on each contig from the reference transcriptome was counted with SAMtools [38]. Count normalization was performed by estimating the normalization factor by the median of scaled counts. The final contingency table was obtained using a Perl custom script and imported in R v 2.11.0 [39]. No and low expressed contigs (≤2 reads aligned found in both SLAP and FLAP) were preliminary filtered, excluding 45% of low expressed contigs and keeping 42,645 contigs for the statistical analysis. Differentially expressed contigs between FLAP and SLAP were identified using the DESeq package v. 1.8.3 [40], applying a method based on the negative binomial model implemented in R software. The estimation of the dispersion parameter was conducted by assuming a local linear relationship between variance and mean expression levels. DESeq provided *p*-values for an exact test which were next adjusted to *p*-value FDRs for multiple testing with the Benjamini–Hochberg procedure [41]. Transcripts were considered differentially expressed for an FDR < 0.10. Annotation of the differentially expressed contigs between FLAP and SLAP was performed using BLASTX (BLASTX 2.2.23) against the NCBI nonredundant database using an E-value cutoff of 10^−5^.

### 2.4. Validation of RNA-Seq Data by Reverse Transcription Quantitative PCR

Total RNAs from three different pools of antennae and palps collected on both starved and fed larvae from different generations (biological replicates; *n* = 3 for each condition) were extracted with the RNeasy MicroKit (Qiagen, Hilden, Germany), which included a DNase treatment. cDNAs were synthesized using the advantage RT-for-PCR Kit (Clontech, Mountain View, CA, USA). Gene-specific primers for three *S. littoralis* regulated contigs (c6022, encoding a putative odorant-degrading enzyme: ODE; c997 and c65324, both encoding candidate chemosensory proteins: CSPs [32]) and for the reference gene *rpL8* were designed using Primer3Plus v. 2.3.0 [42] (Table S1), yielding PCR products ranging from 100 to 200 bp. The qPCR mix was prepared in a total volume of 12 μL with 6 μL of iQ SYBR Green Supermix (Bio-Rad Laboratories, Hercules, CA, USA), 3 μL of diluted cDNA (or water for the negative control or RNA for controlling the absence of genomic DNA), and 200 nM of each primer. The qPCR assays were performed using a CFX96 Touch™ Real-Time PCR Detection System (Bio-Rad). The PCR program began with a cycle at 95 °C for 3 min, followed by 40 cycles of 10 s at 95 °C and 30 s at 60 °C. To assess the specificity of the PCR reactions, a dissociation curve of the amplified products was performed by gradual heating from 65 °C to 95 °C at 0.5 °C/s. Standard curves were generated by a 5-fold dilution series of a cDNA pool evaluating primer efficiency E (E = 10^(−1/slope)^). For each case, the presence of only one amplified product was verified. All reactions were performed in duplicate on the three biological replicates. Expression levels between chemosensory tissues from fed and starved larvae were calculated relative to the expression of the reference gene according to Pfaffl (2001) [43].

## 3. Results and Discussion

### 3.1. Starvation Enhanced Olfactory Behavior in S. littoralis Larvae

We first investigated the effect of 24 h starvation on the olfactory behavior of fourth instar *S. littoralis* larvae using a Petri dish assay (Figure 1a). The preference index of starved larvae toward a piece of food compared to the empty side of the dish significantly differed from that of fed larvae (Figure 1b; *p* < 0.001, Mann–Whitney test), revealing that starvation enhanced olfactory attraction to food, although we cannot exclude a group effect. This result confirms earlier observations on this species [44,45].

### 3.2. Starvation Leads to Up- and Down-Regulation of 81 Transcripts in Larval Chemosensory Organs

**Figure 2:**
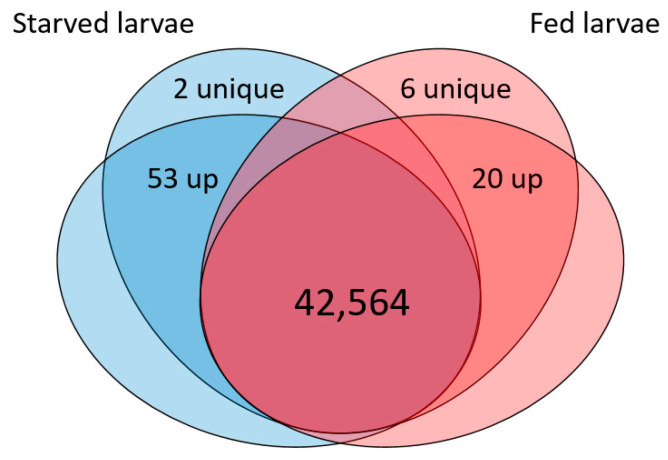
Venn diagram showing the expression of transcripts in the starved (blue) and fed larvae (red) conditions. The number of up-regulated transcripts are indicated as “up”. Numbers of transcripts expressed in only one condition are indicated as “unique”.

Behavioral and physiological evidence of an increase in olfactory sensitivity upon starvation has been reported in both vertebrates and insects [4,16,17,20], but the molecular mechanisms leading to this enhanced sensory sensitivity remain elusive. Here, we used the reference transcriptome we previously established in *S. littoralis* [32] to profile gene expression in antennae and maxillary palps of fed (“FLAP” sample) and 24 h starved (“SLAP” sample) larvae (dataset accession numbers are available in Section 2.2). Short Illumina reads from each condition were mapped on the reference and counted, after which the numbers were compared for each mapped contig (Table 1). After data filtering and median normalization, we observed a high correlation between FLAP and SLAP datasets (*r* = 0.93), indicating that the expression of the large majority of contigs remained stable irrespective of the feeding status. A list of 81 contigs differentially expressed was determined by applying an FDR cutoff of 0.1 (Figure 2). Fifty-five contigs were up-regulated in SLAP with fold change values from 5.7 to infinity (i.e., no expression in fed larvae) (list in Table S2, sequences in File S1), 47% of which had an expression level more than 10-fold found in fed larvae. On the other hand, 26 contigs were downregulated in SLAP, with fold change values from 5.6 to infinity (i.e., no expression in starved larvae) (list in Table S3, sequences in File S2) and, impressively, all contigs but one exhibited large variations (i.e., >10-fold).

### 3.3. Starvation Affects Different Biological Functions

Differentially expressed contigs were annotated by homology search against the NCBI NR database. Among the 81 contigs, 15 had no hit or hits with hypothetical proteins of unknown function. Highly up-regulated sequences under starvation (more than 10-fold) consisted of contigs encoding nine cytochrome P450 enzymes (known to be involved in response to stress, detoxification, or metabolism), 17 takeout-like proteins, a juvenile hormone-binding protein (JHBP, JH being involved in maintaining larval development), two JH acid methyltransferases, two chemosensory proteins (CSPs), a viral enzyme (integrase), a farnesyl diphosphate synthase (providing cells with metabolites), and a putative CRAL/TRIO domain-containing protein (involved in interaction of retinoids with visual cycle enzymes [46]) (Table S2). Highly down-regulated sequences under starvation (more than 10-fold) consisted of contigs encoding proteins involved in host defense and response to pathogens, such as chitin-binding proteins [47] (seven contigs) and REPAT proteins (two contigs) [48], enzymes involved in digestion/immune response such as a protease (trypsin-like serine protease) and a lipase, five takeout-like proteins, and a cuticle protein (Table S3).

Thus, starvation led to a complex transcriptomic response in the peripheral chemosensory organs of caterpillars involving diverse molecular pathways. Among the transcripts whose expression level was related to the nutritional status, some belong to gene families that one would expect to be controlled by feeding, such as genes involved in nutrient metabolism (protease and lipase) and in food search (foraging behavior, olfaction, and vision, although *caterpillar vision* is poor, as they can only differentiate dark from light). Others are assigned to functions that are not obviously related to starvation, such as larval development or diapause (JH, cuticle proteins, and cytochrome P450s, with the last being known to be involved in the synthesis and degradation of ecdysteroids and hormones), pathogen infestation (e.g., REPAT), and responses to stress and/or xenobiotics (such as cytochrome P450s).

Similar pathways have previously been observed to be regulated in studies conducted in *Drosophila*. At the whole organism level, Zinke et al. [24] evidenced modulation of genes possibly involved in cell growth in the face of nutrient deprivation. In *Drosophila* heads, Fujikawa et al. [25] evidenced regulation of the immune response, a phenomenon further observed in all tissues examined by Farhadian et al. [16]. On the contrary, while Ko et al. evidenced regulation of many receptors for biogenic amines, peptides, and neurotransmitters in the antennae of adult *D. melanogaster* upon starvation (including sNPF receptor, dopamine 2-like and dopamine-ecdysone receptors, serotonin 2A receptor, and the GABA-B receptor type 1 [27]), we did not find such receptors to be regulated in larval antennae and maxillary palps, even though our reference transcriptome contained many of these receptors, e.g., octopanime/tyramine, cardioacceleratory peptide, moody, and somatostatine receptors [32].

When precisely focusing on *Drosophila* chemosensory organs, Farhadian et al. [16] revealed down-regulation of transcripts involved in numerous defense and immune processes, as well as sensory responses, in particular to odor and pheromone, and up-regulation of genes involved in different metabolic processes and responses to extracellular stimuli [16].

Looking in detail at specific genes, previous studies reported contradictory results on the regulation of *sNPFR1* in the fly antennae; whereas Root et al. [22] and Ko et al. [27] found strong up-regulation of this gene after fasting; Farhadian’s study [16] failed to find any regulation of this gene, probably because of different temporal timings in the experiments. In *S. littoralis*, the expression of this gene in chemosensory organs could not be examined because no sNPFR-encoding sequence could be identified in the reference transcriptome [32]. Interestingly, we could identify a transcript encoding the sNPF peptide precursor (Slit_qualite_c16336) in the reference transcriptome, and its expression was not impacted by starvation. Since sNPF and its receptor usually fluctuate together, this result suggests that sNPF is not regulated under our conditions, as in Farhadian et al. [16]. *Takeout* genes also revealed discordant results from previous studies conducted in *Drosophila*. These genes encode putative juvenile hormone-like-binding proteins and are expressed by the fat body, similarly to leptin in vertebrate adipose tissues. Very little is yet known on the function of *takeout* in Lepidoptera, but a link between circadian rhythm and feeding behavior in *Drosophila* has been described [49], while it has been proposed to participate in food intake regulation, with increased expression upon starvation [16,28]. While almost all *S. littoralis takeout*-*like* contigs (17 out of 22) appeared to be up-regulated under starvation in our RNA-seq study (Table S2), a few (five contigs) also appeared to be down-regulated (Table S3). A deeper study of these sequences revealed that the 17 up-regulated ones corresponded to at least two different takeout-like proteins, whereas the five downregulated ones could be translated to at least three different takeout-like proteins. This suggests a complex network of behavioral regulation via such proteins.

### 3.4. Starvation Modulates Peri-Receptor-Encoding but Not Chemosensory Receptor-Encoding Transcripts

Odorant receptors (ORs) and ionotropic receptors (IRs) are key proteins in the peripheral olfactory detection process. They are both expressed at the membrane of ORNs, and, upon olfactory ligand binding, they generate an electrical signal that is transmitted to the brain [50,51,52]. We did not evidence any OR IR transcripts to be modulated 24 h after starvation. Accordingly, two studies based on microarray analyses conducted on *D. melanogaster* did not reveal any chemosensory receptors as up- or down-regulated upon starvation [16,25], not even 48 h post fasting [16]. On the contrary, OR expression modulation has been demonstrated after a blood meal in the mosquito *Anopheles gambiae*, with down-regulation of an OR [10]. Thus, depending on the species considered, hunger and satiety may not lead to the same transcriptional response, at least for ORs. In another RNA-seq study comparing the antennal transcriptomes of *A. sinensis* females 5 h after being fed on blood or on sugar, the authors reported no change in the expression of ORs. However, three CSPs and one OBP were found to be overexpressed following the blood meal, while one IR transcript and one GR transcript were found to be overexpressed after the sugar meal [53].

Interestingly, we observed several up-regulated CSPs 24 h after starvation (Table S2). Together with OBPs, these proteins are thought to bind and transport volatile ligands through the sensillum lymph to the chemosensory receptors [54], among other functions [55]. Whereas we found some CSPs as being up-regulated upon starvation, other previous studies demonstrated down-regulation of OBPs in both vertebrates [56] and *Drosophila* [16,25]. Although these results appear contradictory, they could be explained by the respective functions of the regulated binding proteins. Indeed, one of the *Drosophila* downregulated OBP genes, OBP99b, is in fact male-enriched and, thus, supposed to play a role in reproduction rather than food search. OBP99b down-regulation upon starvation suggests that this is a candidate gene mediating a possible tradeoff between starvation resistance and reproduction [25].

In our study, we also evidenced the modulation of one esterase and numerous P450s. In *S. littoralis*, some antennal esterases have been proven to degrade odorants and pheromone components and, thus, to act as odorant-degrading enzymes (ODEs) [57,58]. Some P450s also act as ODEs in insects [59], and numerous antennal P450s have been described in *S. littoralis* [60].

Taken together, our results suggest that the increased olfactory capacities observed at the peripheral level in insects under starvation would be mainly due to increased expression of genes involved in peri-receptor events (OBPs, CSPs, and ODEs) rather than chemosensory reception events (ORs and IRs). A possible scheme would be that starvation induces increased olfactory sensitivity in insects by increasing the odorant access to the receptor (OBP/CSP expression) and the odorant clearance (ODE expression) close to the ORs. In these conditions, ORNs would be able to detect odorants in lower concentrations than during feeding conditions. Further electrophysiological recordings of larvae ORN responses to food could investigate this hypothesis, although such recordings are challenging to perform on caterpillar antennae.

### 3.5. qPCR Validates the RNA-Seq Expression Profiling

To validate the accuracy of the expression profiling approach, we used RT-qPCR on a selected set of genes. We focused on three candidate olfactory genes encoding one ODE (esterase, c6022) and two CSPs (c997and c65324) whose expression was regulated by starvation. Their relative expression levels in larval antennae and palps were measured relative to the reference gene *rpl8* and compared between starved and fed larvae (Table 2). All three transcripts were found to be up-regulated under starvation, as in the RNA-seq analysis (Table 2). In addition, the maximum and the minimum fold changes were obtained for the same transcripts in both methods, confirming the robustness of the RNA-seq results. Interestingly, the fold changes appeared to be systematically overestimated (2-fold) in RNA-seq analyses compared to the qPCR measurements for all the transcripts studied. Such over- or under-estimation of gene expression in RNA-seq has already been observed, depending, for instance, on the normalization process [61] or the depth of the sequencing [62]. Nevertheless, whereas all studies performed before mainly focused on *Drosophila*, we demonstrated in this study the possibility to use RNA-seq for the transcriptome profiling of starvation in a species for which a genome is not yet available.

## 4. Conclusions

Whereas it has been demonstrated that the olfactory plasticity observed in fasted animals results from enhanced presynaptic activity in the antennal lobes mediated via the short neuropeptide F [22] and possibly insulin and GABA signaling [23], we show here that the nutritional status clearly impacts peri-receptor events in the olfactory detection process. In addition to chemodetection modulation, starvation induces a complex transcriptional response in caterpillar peripheral chemodetection organs. The coordinated regulation of transcripts possibly involved in the defense response to stress and/or detoxification, immune response, energy metabolism, chemical senses, and foraging we evidenced in these organs, reflects the insect global strategy for surviving starvation, reallocating resources where needed. Some of the genes we found modulated by fasting may represent novel pathways that regulate feeding behavior in caterpillars. To confirm their role in hunger regulation, functional studies are now needed, which may highlight new targets to fight against the appetite of these voracious animals.

## Figures and Tables

**Table 1:** Summary of Illumina data. FLAP: fed larvae antennae and palp dataset, SLAP: 24 h starved larvae antennae and palp dataset.

	FLAP	SLAP
Number of raw reads	1,947,899	2,389,809
Number of processed reads	1,807,931	2,171,664
Number of mapped reads	1,513,384	1,813,092

**Table 2:** Real-time RT-PCR (qPCR) quantification of expression levels of selected chemosensory genes and comparison with their RNA-seq fold change. qPCR expression levels are expressed relatively to the rpl8 reference gene. N = 3. ODE: Odorant-degrading enzyme. CSP: Chemosensory protein.

Gene Name and ID	qPCR Relative Expression Level in Fed Larvae	qPCR Relative Expression Level in Starved Larvae	qPCR Relative Fold Change (Starved/Fed)	RNA-Seq Fold Change (Starved/Fed)
ODE (c6022)	1.12 × 10^−3^ ± 3.85 × 10^−4^	5.09 × 10^−3^ ± 2.77 × 10^−3^	3.23 ± 1.00	6.00
CSP (c997)	0.06 ± 0.03	0.29 ± 0.20	6.98 ± 2.33	9.14
CSP (c65324)	0.13 ± 0.07	0.59 ± 0.33	5.42 ± 2.31	11.34

## Data Availability

The RNA-seq data generated in this study were deposited in GenBank (BioProject) under the accession numbers SAMN01908929 (fed *S. littoralis* larvae) and SAMN01908927 (starved *S. littoralis* larvae).

## Supplementary Materials

The following are available online at https://www.mdpi.com/article/10.3390/insects12070573/s1, File S1: Fast sequences of up-regulated contigs after 24 h of starvation, File S2: Fast sequences of down-regulated contigs after 24 h of starvation, Table S1: Primers used in RT-qPCR experiments, Table S2: Transcripts with up-regulated expression after 24 h of larval starvation, Table S3: Transcripts with down-regulated expression after 24 h of larval starvation.
