# Supplementary material for: Transcriptome Profiling of Starvation in the Peripheral Chemosensory Organs of the Crop Pest Spodoptera littoralis Caterpillars
Source: Insects. 2021 Jun 23;12(7):573. doi: 10.3390/insects12070573 (PMC8303696; doi:10.3390/insects12070573)
Supplement: Supplementary file 1 [file insects-12-00573-s001.zip › Table S1.pdf]

Table S1: Primers used in RT-qPCR experiments.

Transcripts	Forward primers (5′–3′)	Reverse primers (5′–3′)	Temperatures
c6022	CCGTAGTTTTTCCTGCGGTA	CGCTGATCATTGACTCCAAG	60 °C
c997	CAGAAGGGCAGGGAACTGAA A	TACTTGGCGAGGATCTGCTT	60 °C
c65324	TGTGTCTGACGACCTTCCTG	CTGCTGGTGCCTTACATCAA	60 °C
rpl8	ATGCCTGTGGGTGCTATGC	TGCCTCTGTTGCTTGATGGTA	60 °C
