# Supplementary material for: Transcriptome Profiling of Starvation in the Peripheral Chemosensory Organs of the Crop Pest Spodoptera littoralis Caterpillars
Source: Insects. 2021 Jun 23;12(7):573. doi: 10.3390/insects12070573 (PMC8303696; doi:10.3390/insects12070573)
Supplement: Supplementary file 1 [file insects-12-00573-s001.zip › Table S2.pdf]

Table S2: Transcripts with upregulated expression after 24 h of larval starvation. Fold changes are expressed as fed/starved. "−∞" denotes that the transcript is expressed only in starved larvae. Full sequences are available as Supplementary Materials (File S1).

Transcript ID (According to [32])	Fold Change (Fed/Starved)	Log_2 Fold Change	*p*-Value	Adjusted *p*-Value	BlastX Best Hit
Slit_qualite_rep_c13175	0.000	−∞	1.117716×10^{-16}	4.319302×10^{-12}	hypothetical protein KGM_12805
Slit_qualite_c39402	0.000	−∞	1.283468×10^{-4}	6.438438×10^{-2}	cuticular protein rr-2 motif 70
Slit_qualite_c39105	0.027	−5.233	8.452419×10^{-6}	7.423529×10^{-3}	cytochrome p450
Slit_qualite_rep_c65044	0.041	−4.597	1.364561×10^{-9}	1.318303×10^{-5}	takeout-like
Slit_qualite_rep_c7469	0.044	−4.492	5.645909×10^{-10}	7.272684×10^{-6}	farnesyl diphosphate synthase 3
Slit_qualite_rep_c62831	0.047	−4.420	1.664670×10^{-7}	4.745312×10^{-4}	cytochrome p450 4g4
Slit_qualite_rep_c35117	0.048	−4.379	6.857713×10^{-9}	3.312618×10^{-5}	cytochrome p450 4c3
Slit_qualite_rep_c68996	0.060	−4.070	1.022418×10^{-5}	8.231318×10^{-3}	takeout-like
Slit_qualite_rep_c4859	0.066	−3.911	1.833161×10^{-6}	2.083549×10^{-3}	hypothetical protein KGM_16335
Slit_qualite_rep_c44295	0.069	−3.862	9.436705×10^{-8}	3.646720×10^{-4}	antennal cytochrome p450 cyp4
Slit_qualite_rep_c25310	0.073	−3.779	1.057498×10^{-7}	3.715087×10^{-4}	cytochrome p450 4g15
Slit_qualite_rep_c186	0.078	−3.683	3.173114×10^{-7}	5.839134×10^{-4}	antennal cytochrome p450 cyp4
Slit_qualite_rep_c3543	0.081	−3.623	2.217828×10^{-7}	4.745312×10^{-4}	juvenile hormone acid methyltransferase
Slit_qualite_rep_c5104	0.083	−3.599	3.281553×10^{-6}	3.170309×10^{-3}	integrase core domain protein
Slit_qualite_rep_c20041	0.085	−3.555	5.092912×10^{-7}	8.200437×10^{-4}	juvenile hormone-binding protein precursor-like
Slit_qualite_rep_c45621	0.085	−3.554	2.270225×10^{-7}	4.745312×10^{-4}	cral trio domain-containing protein
Slit_qualite_rep_c6859	0.086	−3.536	9.752707×10^{-6}	8.193122×10^{-3}	takeout-like
Slit_qualite_rep_c63677	0.087	−3.529	5.266288×10^{-6}	4.732801×10^{-3}	chemosensory protein
Slit_qualite_c63585	0.087	−3.519	1.088761×10^{-5}	8.586549×10^{-3}	juvenile hormone acid methyltransferase
Slit_qualite_rep_c65324	0.088	−3.504	2.227229×10^{-7}	4.745312×10^{-4}	chemosensory protein
Slit_qualite_rep_c68153	0.089	−3.485	2.671096×10^{-6}	2.716364×10^{-3}	takeout-like
Slit_qualite_rep_c61187	0.094	−3.415	2.146711×10^{-5}	1.481384×10^{-2}	uncharacterized protein
gi|300739502|gb|FQ020193.1|FQ020193	0.094	−3.411	7.331637×10^{-7}	1.089707×10^{-3}	cytochrome p450 4g15
gi|300757879|gb|FQ022915.1|FQ022915	0.094	−3.408	2.301403×10^{-6}	2.470429×10^{-3}	antennal cytochrome p450 cyp4
Slit_qualite_rep_c2094	0.095	−3.401	4.587191×10^{-7}	8.057610×10^{-4}	cytochrome p450 cyp305b1 precursor
Slit_qualite_rep_c64412	0.098	−3.345	8.596635×10^{-7}	1.140359×10^{-3}	takeout-like
Slit_qualite_rep_c56372	0.102	−3.295	8.061075×10^{-7}	1.140359×10^{-3}	takeout-like
Slit_qualite_rep_c997	0.110	−3.187	1.612621×10^{-6}	1.947441×10^{-3}	chemosensory protein
Slit_qualite_rep_c66981	0.110	−3.181	1.784234×10^{-6}	2.083549×10^{-3}	cytochrome p450 4g15
Slit_qualite_rep_c68492	0.111	−3.173	1.119743×10^{-5}	8.654267×10^{-3}	takeout-like
Slit_qualite_rep_c52629	0.115	−3.119	2.578112×10^{-6}	2.692664×10^{-3}	takeout-like
Slit_qualite_rep_c20865	0.115	−3.117	1.004790×10^{-5}	8.231318×10^{-3}	cytochrome p450 4g15
Slit_qualite_rep_c5736	0.121	−3.049	3.690441×10^{-6}	3.478376×10^{-3}	antennal cytochrome p450 cyp4
Slit_qualite_c25137	0.122	−3.041	1.189536×10^{-4}	6.129124×10^{-2}	no hit
Slit_qualite_rep_c46060	0.122	−3.036	5.124890×10^{-6}	4.715387×10^{-3}	no hit
Slit_qualite_rep_c64392	0.126	−2.992	9.715429×10^{-6}	8.193122×10^{-3}	takeout-like
Slit_qualite_rep_c2157	0.139	−2.850	1.607494×10^{-5}	1.172075×10^{-2}	chemosensory protein
Slit_qualite_rep_c54066	0.142	−2.819	2.327763×10^{-5}	1.578142×10^{-2}	takeout-like
Slit_qualite_rep_c64822	0.144	−2.794	9.689545×10^{-5}	5.201717×10^{-2}	takeout-like

Slit_qualite_rep_c8209	0.146	−2.780	1.883105×10^{-5}	1.323104×10^{-2}	takeout-like
Slit_qualite_rep_c51949	0.150	−2.737	3.435862×10^{-5}	2.212924×10^{-2}	no hit
Slit_qualite_rep_c8503	0.151	−2.730	2.737699×10^{-5}	1.824063×10^{-2}	chemosensory protein
Slit_qualite_rep_c60842	0.153	−2.706	1.347835×10^{-4}	6.593131×10^{-2}	short-chain dehydrogenase
Slit_qualite_rep_c8778	0.154	−2.694	7.617360×10^{-5}	4.393511×10^{-2}	tpa: cuticle protein
Slit_qualite_rep_c10509	0.155	−2.689	9.320994×10^{-5}	5.170297×10^{-2}	cytochrome p450 4g15
Slit_qualite_rep_c64361	0.159	−2.649	5.622170×10^{-5}	3.448621×10^{-2}	takeout-like
Slit_qualite_rep_c2	0.161	−2.633	4.169083×10^{-5}	2.641148×10^{-2}	takeout-like
Slit_qualite_rep_c3249	0.161	−2.632	4.833975×10^{-5}	3.012970×10^{-2}	juvenile hormone-binding protein precursor-like
Slit_qualite_rep_c66265	0.162	−2.627	1.079122×10^{-4}	5.635348×10^{-2}	takeout-like
Slit_qualite_rep_c1646	0.169	−2.566	7.354278×10^{-5}	4.393511×10^{-2}	antennal cytochrome p450 cyp4
Slit_qualite_c6022	0.169	−2.566	1.299550×10^{-4}	6.438438×10^{-2}	esterase
Slit_qualite_rep_c1592	0.169	−2.563	7.534025×10^{-5}	4.393511×10^{-2}	3-hydroxy-3-methylglutaryl-synthase
Slit_qualite_rep_c57132	0.170	−2.560	8.824547×10^{-5}	5.014938×10^{-2}	takeout-like
Slit_qualite_rep_c65224	0.171	−2.549	7.491208×10^{-5}	4.393511×10^{-2}	takeout-like
Slit_qualite_rep_c64559	0.176	−2.508	9.365511×10^{-5}	5.170297×10^{-2}	takeout-like
