# Supplementary material for: Transcriptome Profiling of Starvation in the Peripheral Chemosensory Organs of the Crop Pest Spodoptera littoralis Caterpillars
Source: Insects. 2021 Jun 23;12(7):573. doi: 10.3390/insects12070573 (PMC8303696; doi:10.3390/insects12070573)
Supplement: Supplementary file 1 [file insects-12-00573-s001.zip › Table S3.pdf]

Table S3: Transcripts with downregulated expression after 24 h of larval starvation. "∞" denotes that the transcript is expressed only in fed larvae. Full sequences are available as Supplementary Materials (File S2).

Transcript ID (According to [32])	Fold Change (Fed/Starved)	Log_2 Fold Change (Fed/Starved)	*p*-Value	Adjusted *p*-Value	BlastX Best Hit
Slit_qualite_rep_c1364	5.679	2.506	9.691638×10^{-5}	5.201717×10^{-2}	nd1 gene product
Slit_qualite_rep_c14232	10.125	3.340	8.852804×10^{-7}	1.140359×10^{-3}	hypothetical protein ESOG_04481
gi|300756729|gb|FQ020363.1|FQ020363	10.673	3.416	1.536410×10^{-6}	1.915260×10^{-3}	takeout-like
Slit_qualite_rep_c11761	11.250	3.492	2.020365×10^{-7}	4.745312×10^{-4}	pancreatic lipase 3
Slit_qualite_rep_c7966	11.667	3.544	2.258753×10^{-6}	2.470429×10^{-3}	REPAT46
Slit_qualite_c21059	12.500	3.644	1.684614×10^{-5}	1.205560×10^{-2}	hypothetical protein KGM_15902
Slit_qualite_rep_c5564	13.636	3.769	2.074716×10^{-7}	4.745312×10^{-4}	chitin-binding protein
Slit_qualite_c6733	13.750	3.781	9.915927×10^{-5}	5.249193×10^{-2}	hypothetical protein KGM_08533
Slit_qualite_rep_c5152	16.875	4.077	2.946257×10^{-6}	2.919363×10^{-3}	conserved hypothetical protein
Slit_qualite_c12703	17.083	4.095	1.424471×10^{-5}	1.079358×10^{-2}	chitin-binding protein
Slit_qualite_rep_c62894	17.292	4.112	2.333116×10^{-7}	4.745312×10^{-4}	takeout-like
Slit_qualite_rep_c11060	17.716	4.147	6.626990×10^{-9}	3.312618×10^{-5}	takeout
Slit_qualite_c8499	18.233	4.188	2.216975×10^{-9}	1.713455×10^{-5}	conserved hypothetical protein
Slit_qualite_rep_c29519	19.063	4.253	8.361418×10^{-7}	1.140359×10^{-3}	chitin-binding protein
Slit_qualite_rep_c2501	20.000	4.322	5.026199×10^{-7}	8.200437×10^{-4}	uncharacterized protein
Slit_qualite_rep_c30198	21.250	4.409	2.618540×10^{-7}	5.059544×10^{-4}	conserved hypothetical protein
Slit_qualite_rep_c6676	22.750	4.508	3.152956×10^{-8}	1.353809×10^{-4}	chitin-binding protein
Slit_qualite_c17729	33.125	5.050	1.606508×10^{-10}	3.104095×10^{-6}	conserved hypothetical protein
Slit_qualite_c14464	35.000	5.129	1.563870×10^{-7}	4.745312×10^{-4}	chitin-binding protein
Slit_qualite_c28227	37.500	5.229	1.533426×10^{-5}	1.139572×10^{-2}	takeout-like
Slit_qualite_c14915	∞	∞	2.802719×10^{-5}	1.835733×10^{-2}	uncharacterized protein
Slit_qualite_c18122	∞	∞	5.667373×10^{-7}	8.760399×10^{-4}	chitin-binding protein
Slit_qualite_rep_c15771	∞	∞	1.952352×10^{-4}	9.314406×10^{-2}	trypsin-like serine protease
Slit_qualite_rep_c63050	∞	∞	1.952352×10^{-4}	9.314406×10^{-2}	REPAT40
Slit_qualite_c61228	∞	∞	4.954356×10^{-9}	3.190936×10^{-5}	peritrophic membrane chitin-binding protein 1
gi|300752999|gb|FQ030056.1|FQ030056	∞	∞	1.297636×10^{-4}	6.438438×10^{-2}	takeout-like
